# Increased uncoupling protein (UCP) activity in Drosophila insulin-producing neurons attenuates insulin signaling and extends lifespan

**DOI:** 10.18632/aging.100067

**Published:** 2009-07-21

**Authors:** Yih-Woei C. Fridell, Melissa Hoh, Orsolya Kréneisz, Suzanne Hosier, Chengyi Chang, Dane Scantling, Daniel K. Mulkey, Stephen L. Helfand

**Affiliations:** ^1^ Department of Molecular Biology, Cell Biology, and Biochemistry, Brown University, Providence, RI 02912, USA; ^2^ Department of Physiology and Neurobiology, University of Connecticut, Storrs, CT 06269, USA; ^3^ Current address: Department of Allied Health Sciences, University of Connecticut, Storrs, CT 06269, USA

**Keywords:** UCP, Drosophila IIS pathway, glucose homeostasis, IPCs, DILPs, KATP channels

## Abstract

To understand the role of mitochondrial uncoupling protein (UCP) in regulating insulin signaling and glucose homeostasis, we created transgenicDrosophila lines with targeted UCP expression in insulin producing cells (IPCs).  Increased UCP activity in IPCs results in decreased steady state Ca^2+^ levels in IPCs as well as decreased PI3K activity and increased FoxO nuclear localization in periphery.  This reduced systemic insulin signaling is accompanied by a mild hyperglycemia and extended life span. To test the hypothesis that ATP-sensitive potassium (K_ATP_) channels may link changes in metabolic activity (e.g., glucose mediated ATP production or UCP-mediated ATP reduction) with insulin secretion, we characterized the effects of glucose and a specific K_ATP_ channel blocker, glibenclamide on membrane potential in adult IPCs.  Exposure to glucose depolarizes membrane potential of IPCs and this effect is mimicked with glibenclamide, suggesting that K_ATP_ channels contribute to the mechanism whereby IPCs sense changes in circulating sugar.  Further, as demonstrated in mammalian β-pancreatic cells, high glucose initiates a robust Ca^2+^ influx in adult IPCs.  The presence of functional K_ATP_ channels in adult IPCs is further substantiated by in situ hybridization detecting the transcript for the sulfonylurea receptor (Sur) subunit of the K_ATP_ channel in those cells.  Quantitative expression analysis demon-strates a reduction in transcripts for both Sur and the inward rectifying potassium channel (Kir) subunits when IPCs are partially ablated.  In summary, we have demonstrated a role for UCP in adult Drosophila IPCs in influencing systemic insulin signaling and longevity by a mechanism that may involve K_ATP_ channels.

## Introduction

Mammalian mitochondrial uncoupling proteins (UCPs) have been shown to be involved in energy metabolism, β-pancreatic cell function, and aging [1-6].  Located in the inner membrane of mitochondria, these carriers allow leakage of protons into the matrix,thereby disrupting the proton gradient generated by the respiratory electron transport chain and effectively uncoupling substrate oxidation from ATP phosphorylation.  In β-pancreatic cells, insulin secretion depends upon detection of changes in the ATP levels generated by mitochondrial oxidative phosphorylation [7,8].  ed levels of glucose in the β-pancreatic cells cause an increase in the ATP/ADP ratio, leading to closure of the ATP-dependent potassium (K_ATP_) channel, plasma membrane depolarization, opening of the voltage-gated calcium channel, calcium influx, and insulin secretion.  Consistent with this paradigm, elevated UCP2 activity in β-pancreatic cells that should lead to a decrease in the ATP/ADP ratio has been shown to have a negative effect on glucose-stimulated insulin secretion [2,4,9]. Additional studies including the finding that UCP2 activity is stimulated by glucotoxicity and lipotoxicity in diabetic animal models have established a crucial role for UCP2 in the regulation of insulin secretion and β-cell function [10].

Glucose homeostasis is maintained in a remarkably conserved manner between mammals and fruit flies.  Analogous to the insulin-secreting β-pancreatic cells and glucagon-secreting pancreatic islet a-cells that act in opposition to maintain glucose homeostasis in mammals, fruit flies possess neurosecretory insulin-like peptide-producing cells (IPCs) in the *pars intercerebralis* of the brain and adipokinetic hormone (AKH)-producing corpora cardiaca (CC) cells that function in glucose-sensing [11,12]. Genetic ablation of IPCs in the brain mimics a diabetic phenotype, with increased sugar levels in larval and adult hemolymph associated with growth retardation, developmental delay and reduced fecundity [12,13]. Conversely, targeted ablation of the AKH-producing CC cells renders larvae and adults hypoglycemic [11,14].  Seven*Drosophila* insulin-like peptides (DILPs) have been identified with five of them (DILPs 1-5) showing high homology with their mammalian counterparts [15] whereas a single *Drosophila *AKH peptide has been documented [16,17].  While genetic studies have indicated that adult IPCs are likely the primary endocrine tissue responsible for DILP secretion and signaling, functional evidence as to how adult IPCs sense circulating sugar is lacking and the underlying molecular mechanism(s) responsible for glucose sensitivity are unknown.

In this study, we demonstrate that targeted expression of exogenous UCP in adult IPCs results in attenuated systemic insulin signaling, a mild hyperglycemia and a significant life span extension.  To test whether the mechanism for *Drosophila* insulin secretion, like that found in the b-pancreatic cells, also involves K_ATP_ channels, we show that adult IPCs respond to glucose and sulfonylurea K_ATP_ channel blocker glibenclamide with membrane depolarization whereas adjacent non-IPCs show no discernable response to either agent.  Furthermore, we have detected a robust Ca^2+ ^influx in IPCs in response to glucose exposure.  These electrophysiological recordings are further supported by *in situ* hybridization detecting transcripts for the sulfonylurea receptor (SUR) subunit of the K_ATP _channel in adult IPCs.  Taken together, we have provided strong evidence suggesting that the mechanism for the release of *Drosophila* ILPs, as for insulin secretion in mammalian β-pancreatic cells, involves K_ATP_ channels.

## Results

### UCP expression in adult IPCs does not engender any measurable damage in these neurons

While *Drosophila* IPCs have been shown to regulate DILPs action, the mechanism whereby this is achieved is not understood [12].  We hypothesized that similar molecular events regulating insulin secretion in mammalian β-pancreatic cells may also be involved in glucose sensing and secretion of DILPs in fruit flies.  We therefore predicted that increased mitochondrial uncoupling in IPCs should alter the intracellular events leading to secretion of DILPs via modulation of intracellular ATP/ADP ratio [18].  Adult *Drosophila* IPCs consist of a distinct cluster of 14 medial neurosecretory cells in the *parsintercerebralis* of the brain and can be readily visualized by targeted GFP expression with an IPC-specific, *dilp2* promoter (SI Figure 1A-1B) [12].  Using the UAS-Gal4 system, we targeted two mammalian UCPs, mUCP1 and hUCP2, to fly IPCs. The UAS-*hucp2 *construct has previously been demonstrated to increase physiological mitochondrial uncoupling activity when targeted to the adult fly nervous system [6].  Indirect immunofluorescence studies with an anti-mUCP1 antibody confirmed targeted expression of this protein specifically in the IPCs (SI Figure 1C-1D).  We next confirmed that constitutive UCP expression in IPCs did not cause death or discernable damage to the IPCs.  IPC neurons from flies coexpressing mUCP1 and GFP under the control of the *dilp2*-Gal4 driver were morphologically indistinguishable from those of control flies expressing only GFP in the IPCs (SI Figure 1B, 1E1, and 1E2).  An intact function of IPCs in these flies was further substantiated by finding normal levels of mRNA expression in fly heads for two of the three DILPs (DILP2 and DILP5) that are selectively expressed in the IPCs (SI Figure 4).

### Increased UCP activity in adult IPCs modulates molecular events associated with insulin signaling pathways 

**Figure 1.**
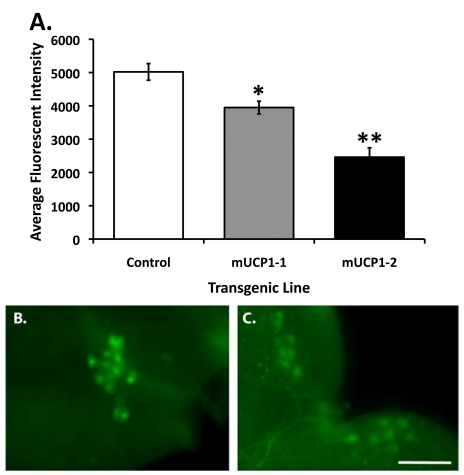
Constitutive UCP expression in adult IPCs results in decreased steady state intracellular Ca ^2+^ levels. (**A**) Under normal growth conditions, the Cg-2 protein in the adult IPCs produces fluorescent signals reflecting a steady state Ca^2+^ flux whereas a 21-51% decrease in average fluorescent intensity is detected in adult IPCs of two *dilp2-*Gal4/UAS*-mucp1*, UAS*-cg-2* co-expressing fly lines (mUCP1-1 and mUCP1-2), consistent with attenuated insulin action. Each bar represents mean + S.E.M.  *p=0.0004, **p<0.0001 (Student's *t*-test).  Shown are average fluorescent signals from IPCs of 10 brains of each line in a representative experiment (see Materials and Methods).  Reproducible results were obtained in three independent experiments.  Representative images of IPCs from a control adult brain isolated from a *dilp2-*Gal4/UAS*-cg-2* fly line (**B**) and an adult brain isolated from a *dilp2-*Gal4/UAS*-mucp1*, UAS*-cg-2* fly line (**C**) are shown.  Images were taken with a 40X objective.  Scale bar, 100 μm.

We investigated whether increased UCP activity in IPCs affects intracellular changes known to be associated with mammalian insulin release.  We began by measuring intracellular Ca^2+^ flux in IPCs.  In order to  do this, we targeted a fluorescent Ca^2+^-binding indicator "camgaroo" (Cg-2) to adult IPCs and tested initially whether these cells responded to food intake with an influx of Ca^2+^as has been shown for larval *Drosophila* CCs in culture and for mammalian β-pancreatic cells [7,11,19].  In adult flies expressing Cg-2 in IPCs that have fasted on water alone, IPCs showed a very low level of Cg-2 fluorescence (SI Figure 2B).  Following re-feeding with glucose or trehalose, two circulating insect sugars, a three-fold increase in Ca^2+^-dependent fluorescence intensity was measured (SI Figure 2A, 2C-2D).  These results demonstrate the ability of adult IPCs to sense extracellular nutrient conditions to increase intracellular Ca^2+ ^concentration, a key step in mammalian insulin release.  Having established that nutrient conditions increase Ca^2+^ concentration in adult IPCs *in vivo*, we next asked whether increased UCP activity in these cells affects intracellular Ca^2+^ levels, using the same Cg-2 reporter fly line.  Under normal growth conditions, we found a 21-51% decrease in steady-state Ca^2+^ levels in IPCs of adult brains isolated from two different mUCP1, Cg-2 co-expressing fly lines as compared to control flies (Figure 1).  Thus, constitutive UCP expression in IPCs results in a decreased steady state intracellular Ca^2+^ flux, a physiological condition consistent with a lowered ATP/ADP ratio and a negative regulation of insulin release.

**Figure 2.**
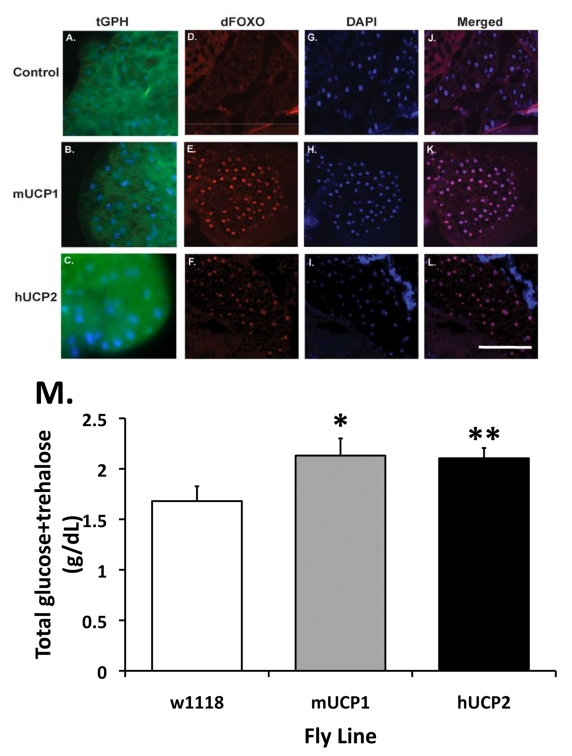
Normal and reduced systemic insulin signaling as reflected by the cellular localization of the PH-tagged GFP reporter protein (tGPH) and dFoxO in fat body cells. (**A-C**) Increased UCP activities in the adult IPCs attenuate systemic insulin signaling events.  In control flies, under normal growth conditions and a full strength PI-3' kinase activity, tGPH is predominantly located at the plasma membrane of each fat body cell (**A**).  (**B-C**) UCP expression in adult IPCs results in a diffused, cytoplasmic distribution of the tGPH protein.  Control: *dilp2*-Gal4, tGPH; mUCP1:* dilp2*-Gal4/UAS-*mucp1*, tGPH; hUCP2: *dilp2-*Gal4/UAS*-hucp2*, tGPH.  (**D-F**) Increased accumulation of dFoxO in the nucleus of pericerebral fat body cells in adult *dilp2-*Gal4*/*UAS*-mucp1* and *dilp2-*Gal4*/*UAS*-hucp2* flies indicates reduced insulin signaling.  Cryosections of adult heads were stained with an α-dFoxO antibody followed by Alexa 568-conjugated secondary antibodies.  A strong nuclear staining of the dFoxO protein was observed in the pericerebral fat body in both *dilp2-*Gal4*/*UAS*-mucp1* (mUCP1, Panel E) and *dilp2-*Gal4*/*UAS*-hucp2* (hUCP2, Panel F) flies but not in *dilp2*-Gal4/*w1118 *(Control, Panel D) flies.  All sections were counter stained with DAPI (Panels G-I) to locate the nucleus of each cell.  Merged images of anti-FoxO staining and DAPI are shown in Panels J-L. (**M**) Elevated levels of fasting circulating sugars are measured in adult *dilp2-*Gal4*/*UAS*-mucp1* and *dilp2-*Gal4*/*UAS*-hucp2 *flies.  An average of 29% increase in circulating sugars measured in 14-day-old *dilp2*-Gal4/UAS-*mucp1* (mUCP1) and *dilp2-*Gal4/UAS*-hucp2* (hUCP2) females as compared to control* dilp2*-Gal4/*w1118 *(w1118) females.  Each bar represents mean + SEM.  N=5-7, *p= 0.046, **p= 0.05 (Student's *t* test).

To understand how systemic insulin signaling may be influenced by increased UCP activity in adult IPCs, we measured the activity of PI-3' kinase, an integral part of the insulin signaling cascade [20]. Responding to the activated insulin receptor, PI-3' kinase generates the second messenger phosphatidylinositol-3,4,5-P3 which in turn interacts with the pleckstrin homology (PH) domain found in several proteins involved in the PI-3' kinase signaling and recruits them to the plasma membrane.  Therefore, membrane localization of PH containing molecules indicates increased PI-3' kinase activity.  A reporter construct consisting of PH-tagged GFP under the control of the ubiquitous tubulin promoter (tGPH) enables monitoring of PI-3' kinase activity downstream of insulin receptor activation [21].  Consistent with the decreased Ca^2+^ levels measured in adult IPCs as the result of increased UCP activity, we detected reduced membrane localization and increased cytoplasmically retained tGPH reporter protein in the insulin-responsive abdominal fat body of adult flies co-expressing tGPH and mUCP1 or hUCP2 under normal feeding conditions (Figure 2B and 2C).  This is in sharp contrast to a predominant plasma membrane accumulation of the tGPH protein in fat body of control flies (Figure 2A), suggesting that systemic insulin signaling is attenuated when UCP is expressed in adult IPCs.  Another functional measure of peripheral insulin signaling is the sub-cellular localization of the *Drosophila* homologue of the mammalian forkhead Box O (FoxO) transcription factor, known to be involved in regulating the insulin signaling pathway [22].  During normal insulin signaling FoxO is phosphorylated and found in the cytoplasm. However, under conditions of decreased insulin signaling, FoxO remains unphosphorylated and localizes predominantly to the nucleus [22,23]. Thus, sub-cellular localization of FoxO reflects the status of insulin signaling in the periphery.  Immunohistochemical analysis of frozen sections of fly heads with an anti-FoxO antibody [22] allowed us to track the sub-cellular localization of dFoxO in the pericerebral fat body of adult UCP-expressing flies.  We found a significant increase in nuclear dFoxO staining of pericerebral fat body cells isolated from UCP-expressing flies as compared to controls (Figure 2D-2F).  Taken together, evaluation of PI-3' kinase activity and dFoxO sub-cellular localization, two independent methods for assessing systemic insulin signaling, confirms that targeted UCP expression in adult fly IPCs reduces systemic insulin signaling.  Having established at the cellular level attenuated insulin signaling, we next asked whether  expression of UCP in adult IPCs also regulates levels of circulating sugars.  When measuring both circulating glucose and trehalose levels in the hemolymph under fasting conditions, we found an up to a 29% increase in circulating sugars in adult flies expressing UCP in their adult IPCs, compared to control flies (Figure 2M).  This finding demonstrates that at the whole animal level, UCP expression in adult IPCs moderately disrupts glucose homeostasis likely as a consequence of decreased secretion of some of the DILPs.

**Figure 3.**
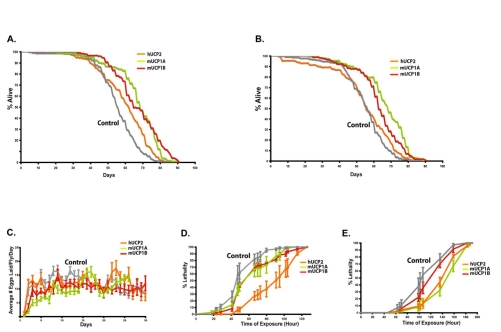
Targeted UCP expression in the adult IPCs extends life span and renders flies stress resistant with normal fecundity. Survivorship curves for female (**A**) and male (**B**) flies are shown.  Two independent trials were performed and results from one trial are shown for one hUCP2 and two mUCP1 lines.  Gray lines are control *dilp2-*Gal4/*w1118* flies; two mUCP1 expressing *dilp2-*Gal4/UAS*-mucp1* lines, mUCP1A and mUCP1B are green and red lines, respectively; and orange lines are *dilp2-*Gal4/UAS*-hucp2*.  Panel A (females) median life spans are 55 for controls and 71, 63, and 65 for three UCP transgenic lines.  Panel B (male) median life spans are 57 for controls and 70, 59, and 59 for three UCP transgenic lines.  Log rank analysis shows an average of 19% increase in median life span in female with targeted UCP expression in the adult IPCs and an average of 10% increase in male (see SI Supplementary Table 1).  (**C**) Female fecundity is similar for flies expressing mUCP1 or hUCP2 in the adult IPCs and control flies. Average number of eggs per day for 20 individual females was determined from daily counts of eggs produced from single mated pairs [6].  (**D**) Transgenic flies with targeted UCP expression in the adult IPCs are resistant to oxidative stress.  Survival during administration of paraquat (20 mM paraquat in 5% sucrose) shows that 10-day-old flies expressing mUCP1 or hUCP2 in the IPCs are more resistant than controls.  (**E**) mUCP1 or hUCP2-expressing flies are resistant to starvation.  Ten-day-old *dilp2-*Gal4*/*UAS*-mucp1 and dilp2-*Gal4*/*UAS*-hucp2 *flies were placed in vials containing water soaked filters and the number of dead flies was counted at noted time intervals.  Three independent assays were performed and a representative experiment is shown for females.  All values are presented as mean ± S.E.M.  Similar differences were seen for males and females.  Each experiment included 8-10 vials with 20 flies in each vial, total of 160-200 flies for each condition.

### Transgenic flies expressing UCP in IPCs are long-lived and stress resistant with unique alterations in *dilp3* expression

By targeting UCP expression to the adult IPCs, we have demonstrated a systemic reduction of insulin signaling activities.  Reduced insulin/IGF-1 signaling has been shown to be a conserved mechanism for life span extension in multiple model organisms [24].  To assess the impact of our transgenic system on longevity, we performed survivorship studies and found that expression of mUCP1 or hUCP2 in adult IPCs extended life span.  Using three different transgenic lines in two independent trials, we observed an average increase in median life span in females of 19% (Figure 3A), and of 10% in males as compared to genetically matched controls (Figure 3B) (SI Supplementary Table 1).  Significantly, this extended longevity does not come with any measurable physiological costs.  As shown in Figure 3C, average egg laying activities were comparable between control and UCP expressing flies, consistent with the view that endocrine manipulations for life span extension are not necessarily associated with compromises in reproduction [24].  In addition, we show that the long-lived UCP transgenic flies are also resistant to oxidative stress (Figure 3D) and starvation (Figure 3E).  Finally, we monitored the physical activity of these transgenic flies and found comparable levels of spontaneous activity between flies having constitutive UCP expression in their IPCs and control flies (data not shown).

**Figure 4.**
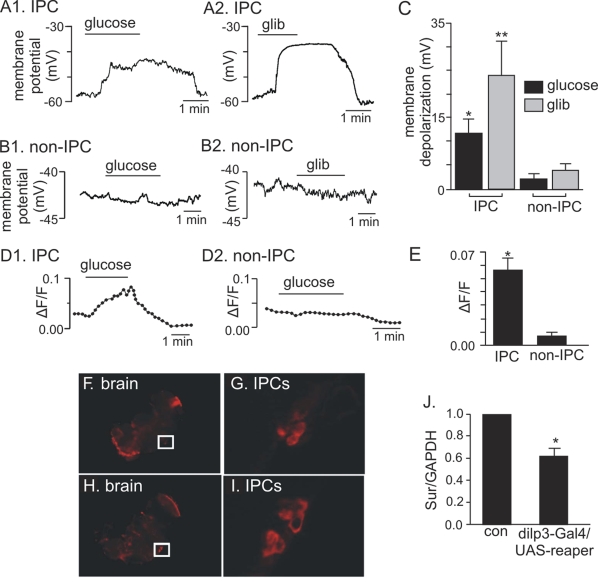
Electrophysiological, Ca ^2+^ influx, and expression evidence of functional K_ATP_ channels in adult IPCs. (**A1-C**) Membrane depolarization of adult IPCs in response to glucose and glibenclamide.  (**A1**) Trace of membrane potential from an adult IPC in the whole brain preparation shows that exposure to high glucose (80 mM) evoked a reversible membrane depolarization.   (**A2)** Trace of membrane potential from an adult IPC shows that exposure to a commonly known K_ATP_ channel blocker, glibenclamide (glib, 20 μM) also evoked a reversible membrane depolarization.  (**B1** and **B2**) traces of membrane potential from adult non-IPCs show that these cells do not respond to glucose or glibenclamide.  (**C**) Average membrane potential response to glucose and glibenclamide of IPCs (N=5) and non-IPCs (N=3).  Glucose (*) and glibenclamide (**) significantly increased membrane potential of IPCs as compared to non-IPCs, *p and **p <0.05 (Student's *t* test).  Each bar represents mean + S.E.M.  (**D1-E**) Glucose-dependent Ca^2+^ influx measured in adult IPCs.  (**D1**) Normalized fluorescence trace (∆F/F) (see Materials and Methods) recorded from an acutely dissociated adult IPC shows that exposure to glucose (80 mM) increased fluorescence intensity, thus indicating an increase in intracellular Ca^2+^.  (**D2**) Normalized fluorescence trace (∆F/F) recorded from an acutely dissociated non-IPC shows that glucose does not increase intracellular Ca^2+^ in these cells.  (**E**). An average of normalized fluorescence intensity in response to glucose demonstrates a significant increase in Ca^2+^ influx recorded from IPCs (N=6) as compared to non-IPCs (N=3).  *P= 0.007 (Student's *t* test).  Each bar represents mean + S.E.M.  (**FI**) *In situ* hybridization of whole mount adult brains demonstrates *dSur *expression in IPCs (F-G) when probed with anti-sense *dSur *probes and *dilp2* expression (**HI**) when probed with anti-sense *dilp2 *probes.  The IPCs marked in squares are shown in panel G for *dSur* signals and panel I for *dilp2 *signals. (**J**) Quantitative real-time PCR analysis reveals an average of 33% reduction in *dSur* transcripts when the IPCs are partially ablated using an IPC-specific driver, *dilp3*-Gal4 to drive the expression of a pro-apoptotic gene, *reaper*.  The housekeeping gene GAPDH was used as the reference gene.  Each bar represents mean + S.E.M.  N=5, *p<0.001 (Student's *t* test). Control: *dilp3*-Gal4/*w1118*.

To understand how energy utilization in these flies was affected, we measured body composition of two major energy stores: glycogen and lipid.  We found an average of 26% increase in glycogen storage in flies having UCP expression targeted to their IPCs (SI Figure 3A).  On the other hand, in contrast to the previous report of a slight elevation (10%) of lipid triglyceride content in animals with ablated IPCs [13], targeted UCP expression in IPCs of adult flies does not result in a noticeable increase in lipid storage (SI Figure 3B).  UCP-induced reduction of insulin secretion, as evidenced by a decrease in Ca^2+^ flux and attenuation of insulin signaling in peripheral fat body, may also have an effect on insulin production.  Interestingly, when the transcript level of the three DILPs found in the IPCs (DILP2, DILP3, and DILP5) was measured, we detected a selective decrease in only *dilp3* mRNA levels in flies expressing mUCP1 or hUCP2 in IPCs whereas both the *dilp2* and *dilp5* mRNA level remained unchanged from control levels (SI Figure 4).

### Electrophysiological and expression evidence for K_ATP_ channels in adult *Drosophila* IPCs

The key component in regulating Ca^2+^ influx into the mammalian β-pancreatic cells leading to insulin release is membrane depolarization initiated by inhibition of K_ATP_ channels [25,26]. Prompted by our studies demonstrating a decreased Ca^2+ ^flux in adult IPCs with increased UCP activity, we asked whether K_ATP_ channels are involved in regulating insulin secretion in adult fly IPCs. Whole cell currentclamp recordings from adult IPCs labeled with GFP in the whole brain preparation showed that exposure to glucose depolarized membrane potential from -69 ± 8 mV to -57 ± 9 mV (Figure 4A1).  In the *pars intercerebralis* of the fly brain, glucose-sensitivity appears to be a unique property of IPCs since nearby non-IPCs did not respond to glucose (Figure 4B1).  The effects of glucose on IPCs were mimicked by the sulfonylurea glibenclamide with membrane depolarization from -56 ± 7 mV to -30 ± 3 mV (Figure 4A2).  Conversely, non-IPCs did not respond to glibenclamide (Figure 4B2).  As summarized in Figure 4C, a significant net membrane depolarization of 12 ± 4 mV and 23 ± 9 mV specifically in adult IPCs in response to glucose and glibenclamide, respectively was recorded suggesting that K_ATP_ channels contribute to glucose sensing in these cells.  In addition, preliminary voltage clamp recordings from IPCs suggest that glibenclamide inhibited ~ 30% of total outward current, further suggesting functional K_ATP_ channels in IPCs (Mohammad Shahidullah, Yih-Woei Fridell, and Irwin B. Levitan, unpublished results).  To determine if exposure to glucose also initiates Ca^2+^ influx into adult IPCs, we loaded acutely dissociated adult IPCs with a Ca^2+^-sensitive fluorescent dye (rhod-3) and measured Ca^2+^ influx upon exposure to glucose.  Consistent with our electrophysiological results, glucose evoked a significant Ca^2+^ influx into adult IPCs (Figure 4D1) whereas non-IPCs did not show a Ca^2+^ response to glucose (Figure 4D2).  Finally, quantitative measure-ment demonstrated a large increase in Ca^2+^ influx in response to glucose that was specific to brain IPCs (Figure 4E).  To further support our electrophysiological results indicating the presence of functional sulfonylurea-sensitive K_ATP_ channels in adult IPCs, we investigated the expression of the Sur subunit of the K_ATP_ channel in these cells. * In situ* hybridization of whole mount adult brains reveals IPC-specific d*Sur* expression with an anti-sense d*Sur* probe (Figure 4F-4G), but not with a sense d*Sur* probe (data not shown).  In parallel experiments with an anti-sense *dilp2* probe, neurons located in the same position in *parsintercerebralis* stained positive for *dilp2* transcript, confirming IPC-specific *dSur* expression (Figure 4H-4I).  Consistent with this result is the quantitative, real-time expression analysis detecting an average reduction of 33% in *dSur *transcript in the heads of adult flies having partially ablated IPCs [27] (Figure 4J).  And finally, when the expression status of the other subunit of the K_ATP_ channel, Kir was quantified in the heads of adult flies with partially ablated IPCs, an average of 27% reduction in *dKir *transcript was seen (SI Supplementary Figure 5) [28].

## Discussion

 In this report, we have demonstrated that increased UCP activity in adult fly IPCs modulates systemic insulin signaling as measured by both molecular events in the insulin signaling pathway and glucose homeostasis at the organismal level.  As expected, attenuated insulin signaling measured in *dilp2*-Gal4/UAS-*mucp1* and*dilp2*-Gal4/UAS-*hucp2* flies is also associated with a significant life span extension in females and a moderate increase in males [24].  In two peripheral insulin responsive tissues, abdominal and pericerebral fat body, we have demonstrated a reduction in the insulin signaling cascade that is evidenced by decreased PI-3' kinase activity and increased nuclear accumulation of the dFoxO transcription factor.  Both events are associated with reduced insulin signaling predicting decreased circulating DILPs.  Consistent with this notion we show that UCP expression in adult IPCs also leads to moderate fasting hyperglycemia.  While we do not currently know which of the three DILPs produced in the IPCs may be affected in its secretion, we have shown that only the mRNA expression of *dilp3* is significantly reduced as a consequence of increased UCP activity in adult IPCs.  This is in marked contrast to previous reports where a decrease specific to *dilp2* transcripts resulted from modulating insulin signaling through manipulation of the activity of JNK, dFoxO, or *Dm*p53 [23, 29, 30]. Our findings suggest that not only does increased mitochondrial uncoupling in adult IPCs attenuate insulin signaling by potentially decreasing DILPs secretion, but that it may also be involved in a novel transcriptional regulation of *dilp3*.  Thus, modulation of mitochondrial uncoupling in IPCs with our UCP transgenic model system has the potential to uncover novel molecular targets controlled by the neuroendocrine axis that are involved in energy metabolism, glucose homeostasis, and longevity.

A UCP2 activity in mammalian β-pancreatic cells has been implicated in the development of Type II diabetes by altering the ATP/ADP ratio, causing the K_ATP_channel to stay open and leading to decreased insulin secretion [4, 5, 9, 31]. To further strengthen our transgenic system as a genetic model for diabetes, we have taken electrophysiological approaches to understand the mechanism whereby increased UCP activity modulates the release of DILPs in *Drosophila* adult IPCs.  Whole cell current clamp experiments with intact adult IPCs recorded depolarized membrane potential in the presence of extracellular glucose.  Importantly, these effects were mimicked in the presence of the sulfonylurea glibenclamide, a pharmacological blocker of K_ATP_channels.  Under similar nutrient conditions, our real-time Ca^2+^ imaging studies have demonstrated a significant Ca^2+^ influx.  These studies strongly suggest that a conserved cascade of intracellular events leading to β-cell insulin secretion, namely closure of K_ATP_ channels and opening of voltage-gated calcium channels, may also function in adult fly IPCs.  A previous report has noted the absence of expression of both *Sur* and *Kir* in the *Drosophila* larval IPCs [11].  In that report, the expression status of those two genes in the adult IPCs was not determined [11].  In addition to the strong electrophysiological data presented here, we have also demonstrated an expression pattern for both *Sur* and *Kir* by *in situ* hybridization and real-time quantitative RT-PCR that is consistent with the notion of functional presence of K_ATP_channels in adult IPCs.

The *Drosophila* SUR subunit of the K_ATP_ channel was first identified as a major component of AKH secretion in cultured larval CC cells [11].  More recently, in *Drosophila* heart, SUR has been shown to protect against hypoxic stress, electrical pacing induced heart failure, and the flock house virus [27, 32].  Here, in further support of the growing evidence that K_ATP_ channels have evolved to maintain a homeostatic function during both glucose sensing and infection resistance, we present evidence for a potentially important role for K_ATP_ channels in the secretion of the DILPs in adult fly IPCs.  Complementing an already significant body of knowledge on the opposing functions of *Drosophila* IPCs and CC cells in maintaining glucose homeostasis in larval stages [11,12], we have identified molecular events involved in the release of DILPs by adult IPCs, further demonstrating a conserved mechanism for glucose sensing between fruit flies and mammals.

Our studies have revealed the physiological impact of increased mitochondrial uncoupling in the *Drosophila* adult IPCs and the utility of such genetic manipulation to model metabolic disorders such as type II diabetes.  Because aging is one of the risk factors for type II diabetes, it is vitally important to develop model systems to understand the parameters involved in insulin regulation during adulthood.  We show that the genetically malleable model system of adult *Drosophila* is well suited for the study of insulin regulation and it is our hope that a better understanding of insulin secretion by adult fly IPCs will stimulate the development of interventions in the fly that are likely to be relevant to human disease.

## Materials and methods

Generation of mucp1 transgenic flies and double transgenic lines; maintenance of fly stocks. The full-length mouse ucp1 cDNA was reverse transcribed and amplified from the mouse brown adipose tissue RNA (gift of Dr. Leslie Kozak, Pennington Biomedical Research Center).  Subsequent injection of the sequence-verified UAS*-mucp1* construct resulted in several germ line transformants.  Five independent transgenic lines were then back-crossed to the *w1118* stock for 10 generations to achieve the same genetic background as in UAS*-hucp2* flies [6].  Consistent with our hUCP2 studies, two out of five independent UAS*-mucp1* lines (UAS*-mucp1A* and UAS*-mucp1B*) with the highest mitochondrial protein expression (data not shown) also engender the most severe developmental lethality when driven by the ubiquitous *actin-*Gal4 driver (data not shown) [6].  Except for life span, stress resistance and egg laying studies where both transgenic lines were examined, only UAS*-mucp1A* flies were included in the rest of the studies described here.

Double transgenic flies carrying UAS-*mucp1*, tGPH, or UAS-*hucp2*, tGPH insertions were generated through meiotic recombination.  Putative double transgenics selected based on eye color were further confirmed by genomic PCR analysis to ensure the presence of both inserts.  Similar strategies were used to create *dilp2-*Gal4, UAS-*mucp1* flies.  These flies were then crossed to UAS-cg-2 lines (gift of Dr. R Davis, Baylor College of Medicine) for Ca^2+^ measurements.  Despite our repeated attempts, no *dilp2-*Gal4, UAS-*hucp2* flies were obtained.  To partially ablate IPCs, the *dilp3*-Gal4 driver was used to drive the expression of the pro-apoptotic gene, *reaper* in IPCs.

The constitutive *dilp2*-Gal4 driver line was kindly provided by Dr. E. Rulifson (UCSF) and the tGPH line by Dr. B. Edgar (Fred Hutchinson Cancer ResearchCenter, Seattle, WA).  The *dilp3*-Gal4 driver line was obtained from Dr. M. Tatar (Brown University).  The UAS-*reaper* line (5824) was obtained from Bloomington Stock Center.  All fly stocks were maintained in a humidified, temperature-controlledincubator with 12h on/off light cycle at 25°C on standard corn meal/yeast/sucrose/agar diet [6].

Whole brain preparation and electrophysiology. Heads were collected from 10-day old *dilp2*-Gal4/UAS-GFP flies and their brains isolated for digestion in 0.5% Trypsin in HBSS for 7 minutes at room temperature.  Whole brains were stored on poly-L-lysine coated cover slips in hemolymph-like (HL) solution [33] at room temperature until individual cover slips were transferred into recording chamber mounted on a fluorescence microscope (Zeiss Axioskop FS) and continuously perfused with a buffer solution containing in mM: 3 KCl, 101 NaCl, 1 CaCl_2_, 4 MgCl_2_, 1.25 NaH_2_PO_4_, 20.7 NaHCO_3_, 35 sucrose, 5 glucose, bubbled with 5% CO_2_ balance O_2_, pH 7.3.  Individual IPCs were differentiated from nearby non-IPCs by their bright GFP fluorescence; whole cell patch clamp recordings were made in both cell types.  High glucose (80 mM) media was made iso-osmotic by eliminating sucrose and decreasing NaCl.  Whole cell current clamp recordings were made at room temperature using patch electrodes (4-7 mΩ) and Axopatch 200B amplifier (Molecular Devises).  Internal pipette solution contains in mM: 120 KCH_3_SO_3_, 4 NaCl, 1 MgCl_2_, 0.5 CaCl_2_, 10 HEPES, 10 EGTA, 0.3 GTP-Tris, pH 7.2.  Membrane potential was recorded and analyzed using a Digidata 1322A digitizer and pCLAMP 10 software.  All chemicals were purchased from Sigma.

Acute dissociation preparation and real time Ca ^2+^ imaging. To dissociate the cells, *dilp2*-Gal4/UAS-GFP brains were treated as described for the whole brain preparation but incubated in 0.5%Trypsin for 15 min.  Trypsin was then inactivated by adding 5% FBS and the tissue fragments were pelleted and resuspended in modified HL solution containing in mM: 5 KCl, 108 NaCl, 2 CaCl_2_, 8.2 MgCl_2_, 1 NaH_2_PO_4_, 4 NaHCO_3_, 5 HEPES, 10 sucrose, 5 glucose, pH adjusted to 7.3 with NaOH.  The tissue was then titurated and plated on poly-L-lysine coated cover slips at a cell density of ~ 15 brains per cover slip.  Cells were allowed to settle for at least 2h at room temperature before loading with the cell permeable Ca^2+^-dye rhod-3 AM according to manufacturer's instructions (Molecular Probes, Cat. Nr. R10145).  Individual cover slips were submerged in a chamber mounted on a fluorescence microscope (NIKON TE 200) and cells visualized with Nomarski optics with a 40x oil immersion objective; fluorescently labeled IPCs and non-fluorescent control cells were chosen for imaging.

NIS Elements 3.20 software was used to acquire fluorescence images at 0.1 Hz with DsRed filter set (Chroma 42005).  High glucose (80 mM) media was made iso-osmotic by eliminating sucrose and decreasing NaCl.  The response to glucose was normalized by dividing the glucose-induced change in fluorescence (∆F=F_glucose_-F_control_) by control fluorescence (∆F/F).  At the end of each experiment, cell viability was assessed by responsiveness to high Ca^2+^ media (data not shown).

Quantitative real-time expression PCR analysis **. **Total RNA was isolated from heads of 10-day oldfemales using the TRIzol method (Invitrogen), and subsequent cDNAand QPCR experiments were performed as described previously [23].  Two-four independent RNA preparations with triplicates in each QPCR experiment were used to derive the mean ratios of target gene expression against the reference gene GAPDH.  The followingprimers were used: dilp2-F, 5'-AGCAAGCCTTTGTCCTTCATCTC-3';dilp2-R, 5'-ACACCATACTCGCACCTCGTTG-3'; dilp3-F, 5'-AGAGAACTTTGGACCCCGTGAA-3'; dilp3-R, 5'- TGAACCGAACTATCACTCAACAGTCT-3'; dilp5-F, 5'-GAGGCACCT TGGGCCTATTC-3'; dilp5-R, 5'-CATGTGGTGAGATTCGGAGCAA-3'; dSur-F, 5'-GAGCAGGCGACGACAAA-3', dSur-R, 5'-GCCCTC GTATCGCAGACTAAC-3'; dKir-F, 5'-CAGGACAA AGAGCACCAAGGAG-3', dKir-R, 5'- CCAGATGA AGAACAAATCAGAGCC-3'; GAPDH-F, 5'-GAC GAAATCAAGGCTAAG GTCG-3'; GAPDH-R,5'-AATGGGTGTCGCTGAAGAAGTC-3'.

*In situ* hybridization of adult IPCs. *In situ* hybridization of adult brains containing the IPCs was performed as previously described (personal communication with Dr. E Ruflison, UCSF) [11]. To detect *dSur* expression, digoxigenin labeled *dSur* probes were generated by subcloning the cDNA clone SD08664 (Open Biosystems, CA) into a pBluescript vector.  T3- and T7-mediated *in vitro* transcription was performed to generate sense and anti-sense RNA probes.  To use as a positive control, IPC-specific *dilp2* probes were generated in parallel reactions.  The indirect TSA System (PerkinElmer, Wellesley, PA) was used for signal amplification.

Immunofluorescence staining, immunohistochemistry, and fluorescence microscopy. Whole mount fluores-cence experiments with *dilp2*-Gal4/UAS-*mucp1*, UAS-GFP lines were performed to show the integrity of IPCs in the presence of mUCP1 expression [23].  To detect mUCP1 expression in the IPCs of the transgenic *dilp2*-Gal4/UAS-*mucp1* flies, adult brains were dissected and fixed in 4% paraformaldehyde in PBS. The immunofluorescence procedure with an anti-mUCP1 antibody (1:1000, UCP12-A, Alpha Diagnostic) was performed as previously described [30].  For dFoxO subcellular localization, 10-day old adult fly headswere fixed in fresh 4% paraformaldehyde, embedded in tissue freezing medium (TFM, Triangle Biomedical), frozen, cutat 10 μM and mounted on SuperFrost Plus slides (Fisher Scientific).  Slides were washed to remove TFM and stainedwith an anti-dFoxO (1:500, rabbit antiserum) (kindly provided by Dr. O. Puig, University of Helsinki, Finland) following the procedure described by Bauer, et. al. [23].  All imageswere taken using a Zeiss Axiovision Z1 fluorescentmicroscope with identical magnification and exposure time for both control and experimental samples (Thornwood, NY).  For tGPH subcellular localization, adult abdominal fat body tissues were dissectedinto PBS and visualized usingApoTome optics (Zeiss Axiovision Z1 fluorescentmicroscope).  Intra-cellular localization of the fluorescent reporter tGPH in fat body cells under different treatments and genetic backgrounds was scored by involving two people where one person performed the staining and the other read the slides, which have been numerically "blinded" by the first person.

Measuring steady-state Ca ^2+^ concentration in adult IPCs **. **To measure the steady-state intracellular Ca^2+^ concentration in the adult IPCs with the Ca^2+^sensor Cg-2, flies were fasted 12-16 hours and then placed in vials containing 10% glucose or 10% trehalose soaked filters for 30 minutes.  Fly heads were then collected and fixed in 4% formaldehyde/PBS, and brains containing IPCs were dissected for fluorescent measurements.  Ca^2+^-dependent, fluorescent images with identical magni-fication and exposure time for both control and experimental samples were collected by using Axio-vision Apotome microscopy equipped with a CCD camera and quantification of signals was achieved using the Axiovision softwaresuite, Version 4.5 (Zeiss, Inc.).  Arbitrary fluorescent units representing the signal intensity were both generated and analyzed using the Axiovision software [23]. Typically, 8-10 brains/treatment were included in each experiment.  Three independent experiments were performed.  Average signals under each treatment were achieved by quantifying signal intensity of each IPC before calculating the average signal intensity (~112-140 cells/treatment).

Hemolymph collection and carbohydrate measurements. Adult hemolymph was extracted by capillary action after a small puncture to the head capsule near the ocelli.  Flies were reared on standard corn meal/ yeast/ sucrose/agar diet and fasted for 12 hours on 2% agar prior to hemolymph collection.  In each experiment, triplicates of ~40 female flies were used to obtain ~1 ul of hemolymph from each sample.  Multiple [5-7] experiments were performed.  The amount of circulating glucose was measured using the Infinity Glucose Reagent (Sigma) and porcine kidney trehalase (Sigma) was added to convert trehalose to glucose as previously described [11,12].

Glycogen and triglyceride body composition determination. Whole body homogenates from 10-day-old female flies were prepared as described [34].  For each assay, triplicates of 20 μl of homogenate for eachsample were included.  Glycogen content was calculated by subtractingthe total glucose composition without amyloglucosidase digestionfrom the total glucose composition after amyloglucosidase digestion [34].  For, triglyceride measurements, fly homogenates were similarly prepared and subjected to analysis using the triacylglycerolhydrolysis kit (335-UV, Sigma). Three independent experiments were performed.  All results were normalized with fresh fly weight measured immediately before homogenization.

Life span and stress resistance studies. To perform life span studies, homozygous virgins bearing UAS-*mucp1A*, UAS-*mucp1B*, or UAS-*hucp2* transgene and control *w^1118^* female virgins were crossed to homozygous *dilp2*-Gal4 driver males.  The progeny from these crosses were maintained on standard corn meal/yeast/sucrose/agar diet and passed to fresh vials every other day [6].

Both starvation and paraquat resistance assays were conducted as described previously [6].  Briefly, 10-day-old *dilp2*-Gal4/UAS-*mucp1*, *dilp2*-Gal4/UAS-*hucp2* and *dilp2*-Gal4/*w^1118^*flies were placed in vials containing filter paper soaked in water (starvation assay) or a solution of 20 mM paraquat and 5% sucrose (paraquat assay) and the number of dead flies counted every 8-14 hours.  Three independent experiments were performed.  Each experiment used eight to ten vials with 20 males or 20 females in each vial (160-200 males and 160-200 females per experiment).

Female fecundity. Female fecundity was determined from daily counts of eggs produced by 20 individual females in single mating pairs of *dilp2*-Gal4/UAS-*mucp1*, *dilp2*-Gal4/UAS-*hucp2* and *dilp2*-Gal4/*w^1118^*flies fed with regular yeast/sucrose/agar food.  The flies were passed to new vials every day and the number of eggs laid was counted and recorded for the first 24 days of the adult life [6].

Statistical analysis. Statistical analysis for independent life span trials was performed using log-rank test (StatView).  Results for all other assays were analyzed using paired Student's *t* test.

## Supplementary information

Supplementary Table 1 Life spans of mUCP1 and hUCP2 expressing and control flies. Trial 1 and Trial 2 are two independent life span experiments. Genotype: mUCP1A and mUCP1B are two independent *dilp2*-Gal4/UAS-*mucp1* transgenic lines; *hUCP2* is *dilp2*-Gal4/UAS-*hucp2; w^1118^* is *dilp2-Gal4/w^1118^*. Median life spans are calculated by StatView.  % increase is calculated as the percent change between the w1118 flies and UCP expressing flies.  Chi-square and probability (p values) are calculated by log-rank test (StatView).  Maximum life span is calculated as the mean life span of flies remaining at 10% survivorship.  N= number of flies in each life span trial.

Supplementary Figure 1 Exogenous expression of the mUCP1 protein in the adult IPCs does not engender any morphological abnormalities. (**A-B**) Fluorescent images of adult IPCs in the *pars intercerebralis* at low (**A**) and high (**B**) magnification via GFP expression of the *dilp2*-Gal4/UAS-GFP flies. (**C-D**) Immunofluorescent staining with an anti-mUCP1 antibody demonstrating the localization of the mUCP1 protein in the IPCs of the dilp2-Gal4/UAS-mucp1 flies (**D**) but not in the IPCs of the control *dilp2-Gal4/w^1118^* flies (**C**).  (**E1-E2**) Fluorescent images of a representative adult brain isolated from *dilp2-Gal4/UAS-mucp1*, UAS-GFP flies. Images of the same brain on two different focus planes were taken to show a total of 14 IPCs, indistinguishable from those of a control *dilp2*-Gal4/UAS-GFP brain (**B**). Image **A** was taken with a 20X objective whereas images B-E2 were taken with a 40X objective. Scale bars, 100 μm.

Supplementary Figure 2 Changes in intracellular Ca ^2+^ concentration in adult IPCs in response to nutrient conditions. (**A**) A three-fold Increase in fluorescent intensity is measured in adult IPCs producing ″camgaroo″ (Cg-2) in response to glucose or trehalose. Each bar represents mean + S.E.M. (N=3 independent experiments with 8-10 brains analyzed in each experiment). *P < 0.001. (Student's t test). (**B-D**) Representative images of brains of *dilp2-Gal4/UAS-cg-2* flies following 16 hour-fasting (**B**) and refeeding with 10% glucose.  (**C**) or 10% trehalose (**D**) for 30 minutes demonstrate an increase of Ca^2+^-dependent fluorescence in adult IPCs located in the *pars intercerebralis*. All images were taken with a 40X objective. Scale bar, 100 μm.

Supplementary Figure 3 Body composition analysis of adult flies expressing mUCP1 or hUCP2 in IPCs. (**A**) An average of 26% increase in glycogen storage is the result of IPC-specific UCP expression. (**B**) IPC-specific UCP expression does not significantly alter total triglyceride content of the fly. Control: *dilp2-Gal4/w1118*; mUCP1: *dilp2-Gal4/UASmucp1*; hUCP2: *dilp2-Gal4/UAS-hucp2*. Each bar represents mean + S.E.M. N=3. *P=0.005; **p=0.016 (Student's t test).

Supplementary Figure 4 Quantitative real-time RT-PCR analysis reveals a dramatic decrease (70%) in *dilp3* expression in *dilp2*-Gal4/UAS-mucp1 (mUCP1) flies as compared to Control *dilp3-Gal4/w1118* flies. The changes in transcript levels for dilp2 and dilp5 as the result of UCP expression in IPCs are not statistically significant. A 30% decrease in *dilp3* expression in *dilp2*-Gal4/UAS-*hucp2* (hUCP2) females is the average of two independent experiments. The housekeeping gene GAPDH was used as a reference gene. Each bar represents mean + S.E.M except for the *dilp3*/GAPDH value measured in *dilp2*-Gal4/UAS-*hucp2* females where two independent experiments were performed. N=4 independent experiments with 4 separate RNA preparations. In each experiment, each sample was measured in triplicate. *P<0.01 (Student's t test).

Supplementary Figure 5 Quantitative real-time PCR analysis reveals an average of 27% decrease in Kir expression when IPCs are partially ablated using an IPC-specific *dilp3*-Gal4 driver for the expression of a pro-apoptotic gene, *reaper*. The housekeeping gene GAPDH was used as a reference gene. Each bar represents mean + S.E.M. N=4. *P=0.018 (Student's t test).  Control: *dilp3-Gal4/w1118*.
