# Supplementary figures and images for: Increased uncoupling protein (UCP) activity in Drosophila insulin-producing neurons attenuates insulin signaling and extends lifespan
Source: Aging (Albany NY). 2009 Jul 21;1(8):699–713. doi: 10.18632/aging.100067 (PMC2830081; doi:10.18632/aging.100067)

**
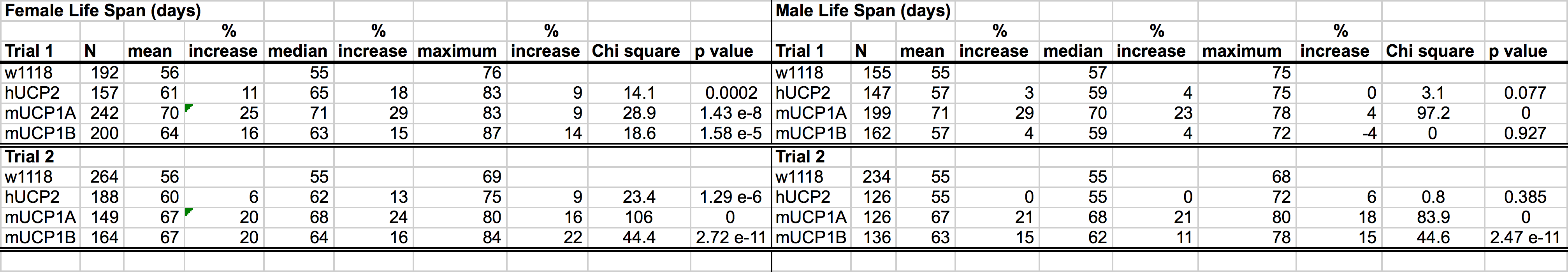
**

Supplement: Supplementary Table 1 — Trial 1 and Trial 2 are two independent life span experiments. Genotype: mUCP1A and mUCP1B are two independent dilp2-Gal4/UAS-mucp1 transgenic lines; hUCP2 is dilp2-Gal4/UAS-hucp2; w1118 is dilp2-Gal4/w1118. Median life spans are calculated by StatView. % increase is calculated as the percent change between the w1118 flies and UCP expressing flies. Chi-square and probability (p values) are calculated by log-rank test (StatView). Maximum life span is calculated as the mean life span of flies remaining at 10% survivorship. N= number of flies in each life span trial. [file aging-01-699-s001.doc]

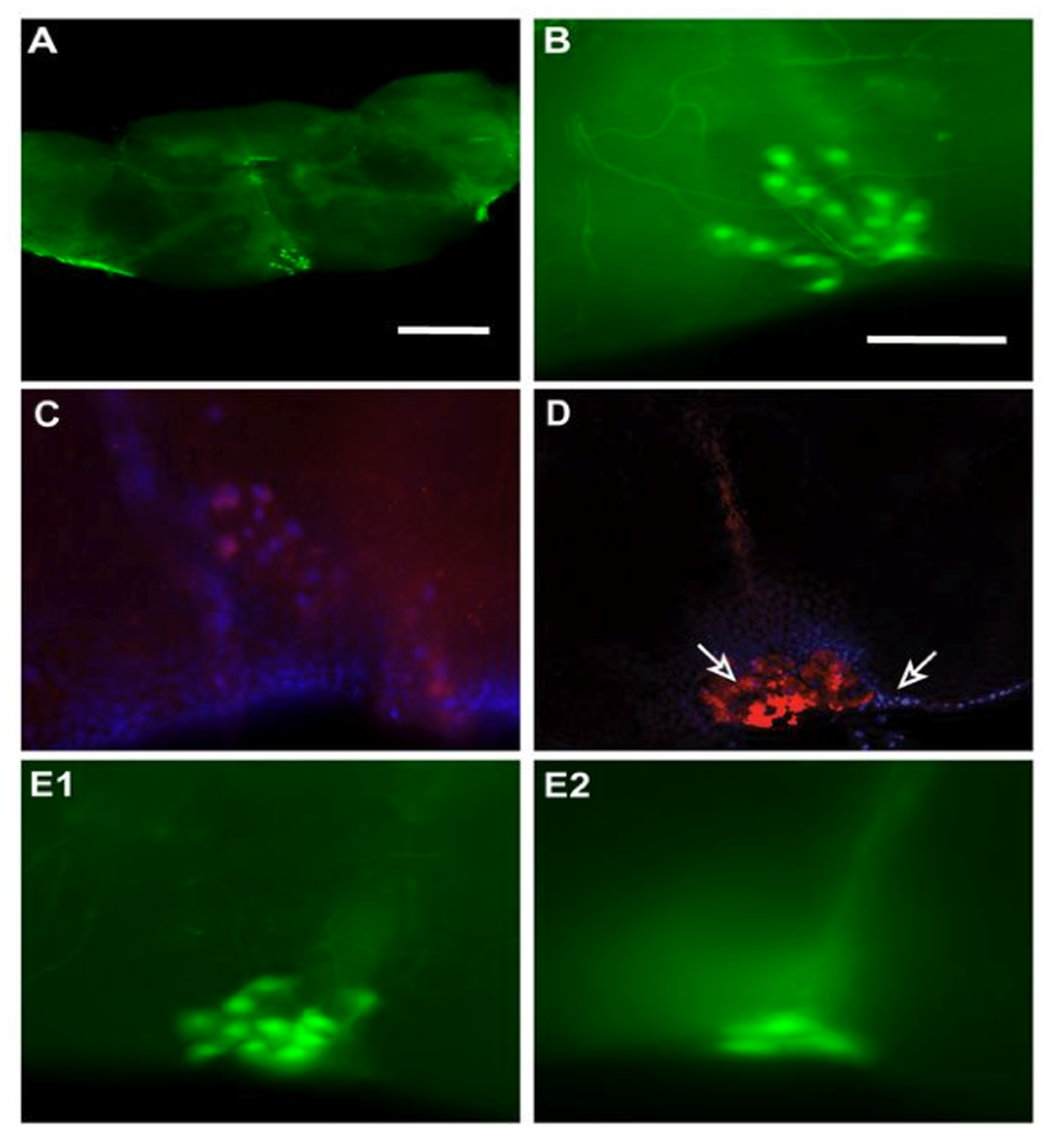

Supplement: Supplementary Figure 1 — (A-B) Fluorescent images of adult IPCs in the pars intercerebralis at low (A) and high (B) magnification via GFP expression of the dilp2-Gal4/UAS-GFP flies. (C-D) Immunofluorescent staining with an anti-mUCP1 antibody demonstrating the localization of the mUCP1 protein in the IPCs of the dilp2-Gal4/UAS-mucp1 flies (D) but not in the IPCs of the control dilp2-Gal4/w1118 flies (C). (E1-E2) Fluorescent images of a representative adult brain isolated from dilp2-Gal4/UAS-mucp1, UAS-GFP flies. Images of the same brain on two different focus planes were taken to show a total of 14 IPCs, indistinguishable from those of a control dilp2-Gal4/UAS-GFP brain (B). Image A was taken with a 20X objective whereas images B-E2 were taken with a 40X objective. Scale bars, 100 μm. [file aging-01-699-s001.tif]

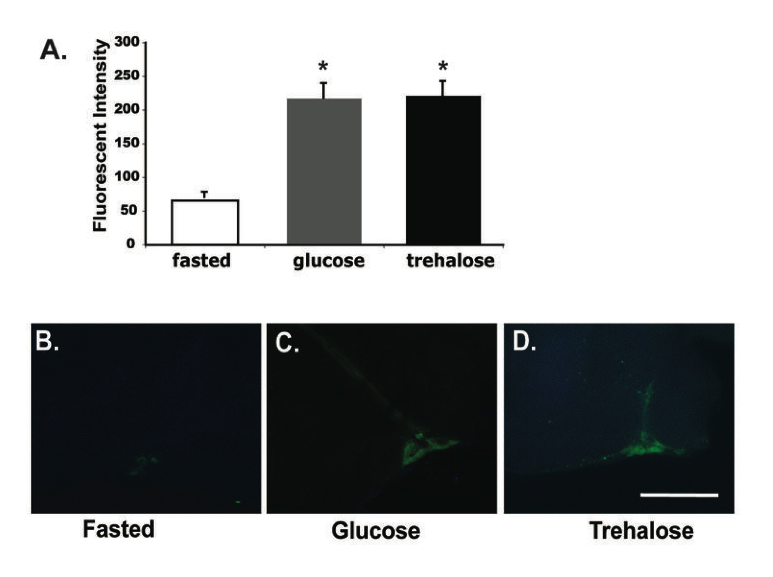

Supplement: Supplementary Figure 2 — (A) A three-fold Increase in fluorescent intensity is measured in adult IPCs producing ″camgaroo″ (Cg-2) in response to glucose or trehalose. Each bar represents mean + S.E.M. (N=3 independent experiments with 8-10 brains analyzed in each experiment). *P < 0.001. (Student's t test). (B-D) Representative images of brains of dilp2-Gal4/UAS-cg-2 flies following 16 hour-fasting (B) and refeeding with 10% glucose. (C) or 10% trehalose (D) for 30 minutes demonstrate an increase of Ca2+-dependent fluorescence in adult IPCs located in the pars intercerebralis. All images were taken with a 40X objective. Scale bar, 100 μm. [file aging-01-699-s002.tif]

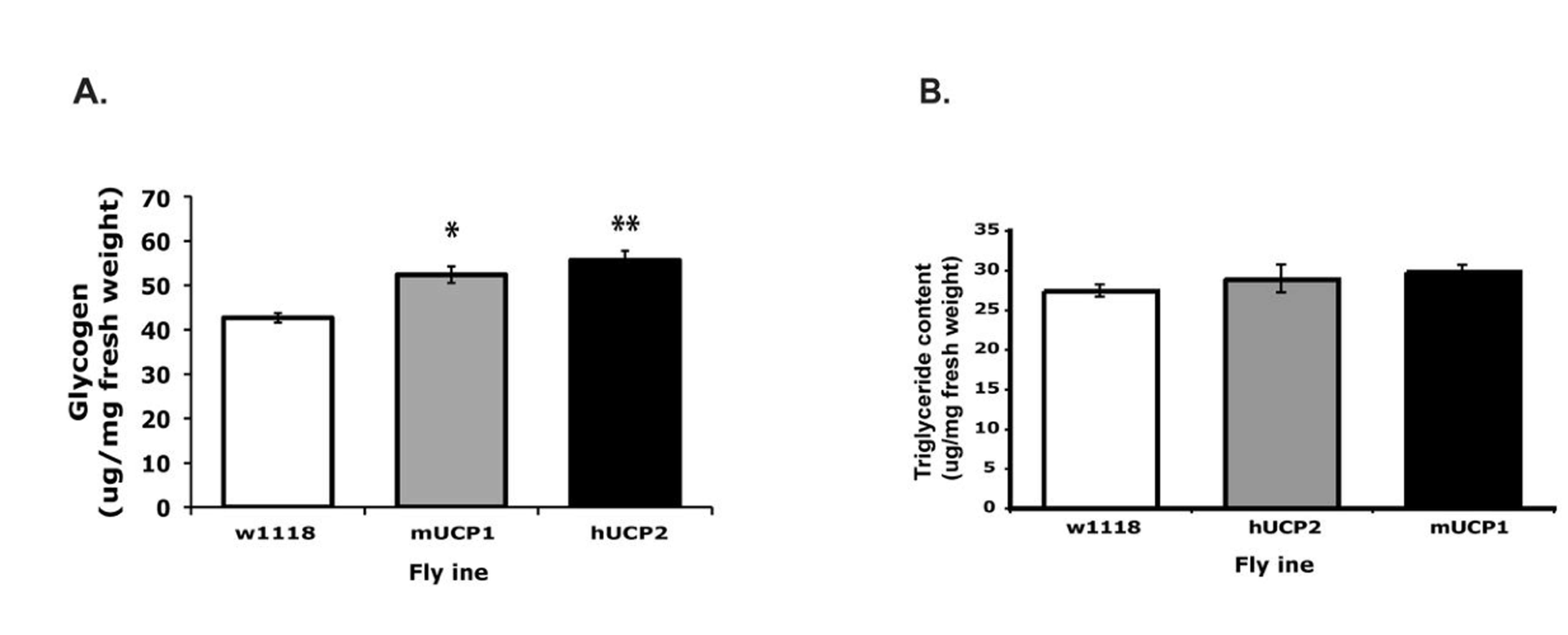

Supplement: Supplementary Figure 3 — (A) An average of 26% increase in glycogen storage is the result of IPC-specific UCP expression. (B) IPC-specific UCP expression does not significantly alter total triglyceride content of the fly. Control: dilp2-Gal4/w1118; mUCP1: dilp2-Gal4/UASmucp1; hUCP2: dilp2-Gal4/UAS-hucp2. Each bar represents mean + S.E.M. N=3. *P=0.005; **p=0.016 (Student's t test). [file aging-01-699-s003.tif]

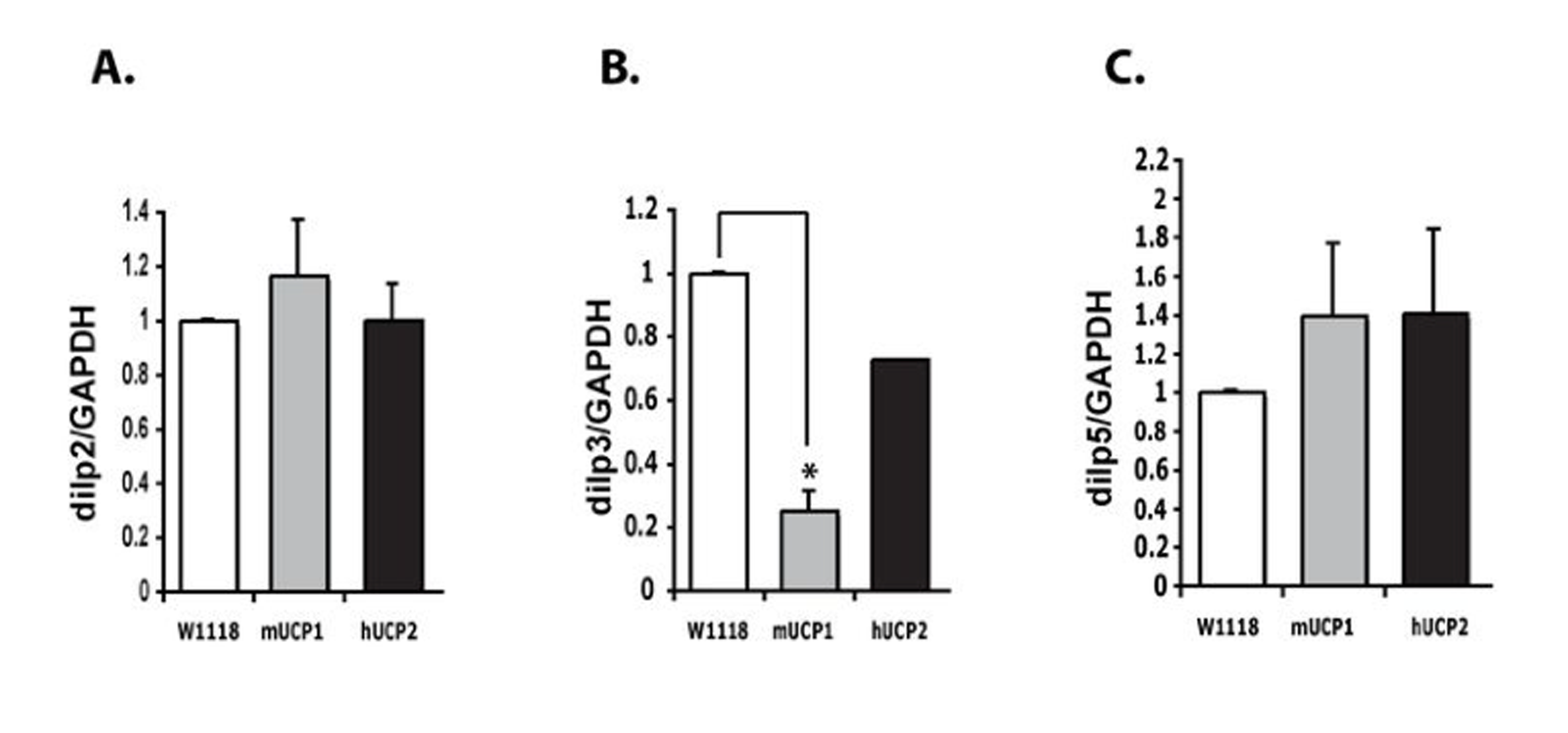

Supplement: Supplementary Figure 4 — Quantitative real-time RT-PCR analysis reveals a dramatic decrease (70%) in dilp3 expression in dilp2-Gal4/UAS-mucp1 (mUCP1) flies as compared to Control dilp3-Gal4/w1118 flies. The changes in transcript levels for dilp2 and dilp5 as the result of UCP expression in IPCs are not statistically significant. A 30% decrease in dilp3 expression in dilp2-Gal4/UAS-hucp2 (hUCP2) females is the average of two independent experiments. The housekeeping gene GAPDH was used as a reference gene. Each bar represents mean + S.E.M except for the dilp3/GAPDH value measured in dilp2-Gal4/UAS-hucp2 females where two independent experiments were performed. N=4 independent experiments with 4 separate RNA preparations. In each experiment, each sample was measured in triplicate. *P<0.01 (Student's t test). [file aging-01-699-s004.tif]

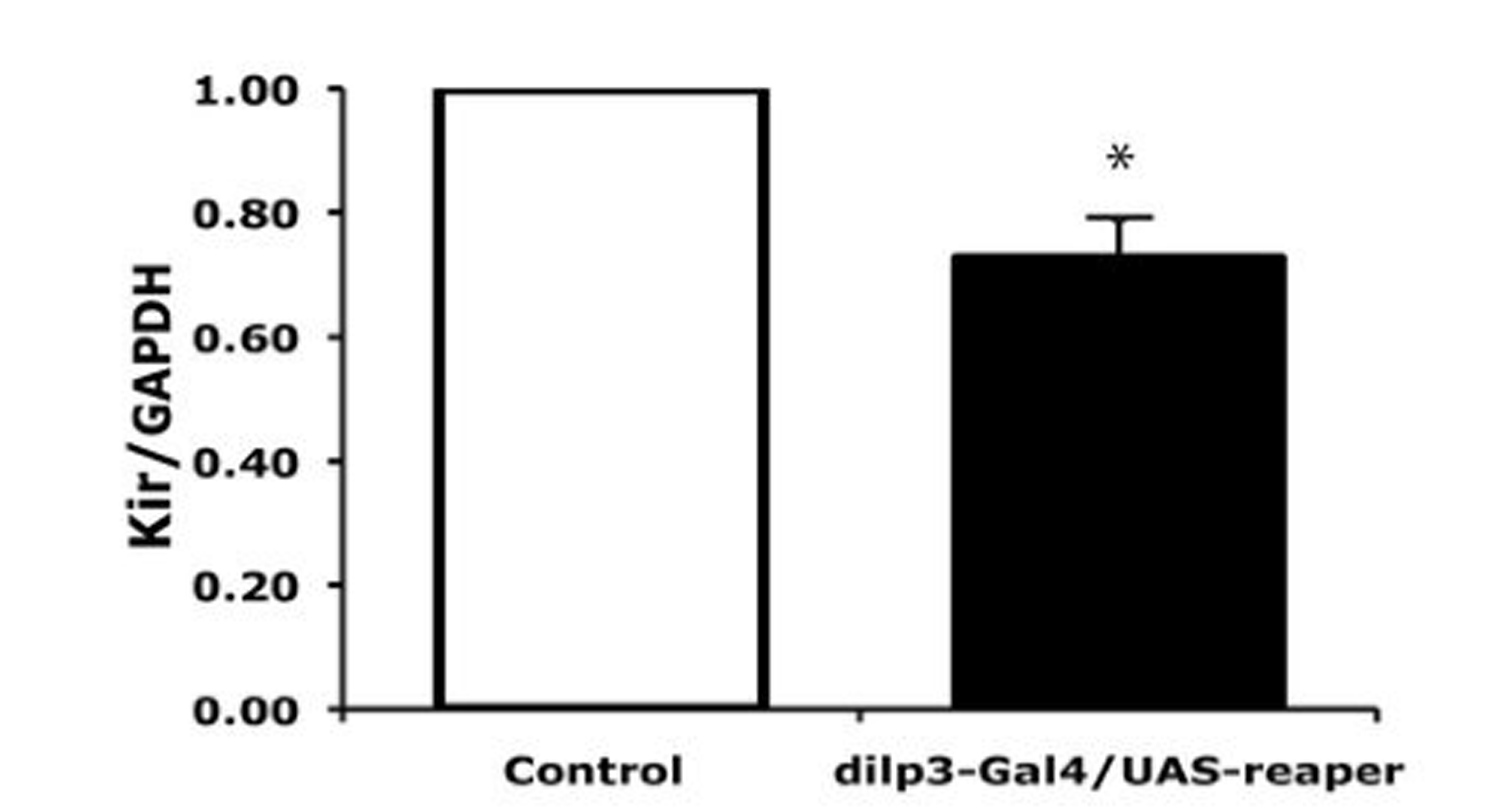

Supplement: Supplementary Figure 5 — Quantitative real-time PCR analysis reveals an average of 27% decrease in Kir expression when IPCs are partially ablated using an IPC-specific dilp3-Gal4 driver for the expression of a pro-apoptotic gene, reaper. The housekeeping gene GAPDH was used as a reference gene. Each bar represents mean + S.E.M. N=4. *P=0.018 (Student's t test). Control: dilp3-Gal4/w1118. [file aging-01-699-s005.tif]
